# Different lymph node staging systems in patients with gastric cancer from Korean: What is the best prognostic assessment tool?

**DOI:** 10.1097/MD.0000000000003860

**Published:** 2016-06-24

**Authors:** Jin Won Lee, Bandar Ali, Cho Hyun Park, Kyo Young Song

**Affiliations:** a Department of Surgery, Chuncheon Sacred Heart Hospital, The Hallym University of Korea, College of Medicine, Chuncheon; b Department of Surgery, Seoul St. Mary's Hospital, The Catholic University of Korea, College of Medicine, Seoul, Korea.

**Keywords:** gastric cancer, lymph nodes, neoplasm staging, stomach

## Abstract

To investigate whether the log odds of positive lymph nodes (LODDS) system is a more accurate prognostic tool than the number-based (pN) or ratio-based (rN) lymph node staging system in Korean patients with gastric cancer (GC).

The LODDS is a recently proposed staging modality in surgical oncology. However, it is unclear whether LODDS is superior to the pN or rN system in terms of predicting the prognosis of GC patients who underwent radical gastrectomy with extended lymphadenectomy and had a greater number of retrieved lymph nodes.

Clinicopathological data from 3929 patients who had undergone curative gastrectomy for GC were reviewed. In addition, overall survival rates according to pN and rN classification stratified by the LODDS were analyzed. A multivariate analysis of survival rate was performed using a Cox proportional hazard model.

pN, rN, and LODDS were significantly correlated with 5-year survival rate. Spearman correlation test showed no correlation between LODDS and number of lymph nodes retrieved. The receiver operating characteristic (ROC) curves showed that the 3 staging systems had comparable prognostic accuracy (*P* < 0.05). Survival analysis according to pN and rN classification stratified by the LODDS staging system demonstrated that LODDS is superior to pN and rN.

The LODDS is independently and significantly associated with the OS of Korean patients with GC, and its prognostic value is superior to that of the other lymph node staging systems in Korean patients.

## 1 Introduction

Gastric cancer (GC) is one of the most common malignancies among all types of solid cancer and the 2nd leading cause of cancer-related death worldwide.^[1]^ The incidence of GC varies geographically; for instance, it is considerably higher in East Asian compared with Western countries. The nodal status is the most important prognostic factor for accurate staging and therapeutic decision making in the management of GC.^[2–4]^ In the 7th edition of the International Union Against Cancer (UICC)/American Joint Committee on Cancer (AJCC) tumor node metastasis (TNM) classification, the pathologic nodal (pN) stage is classified as pN0, pN1, pN2, pN3a, or pN3b according to the absolute number of metastatic nodes.^[5]^ However, there has been consensus of stage migration, also known as the “Will Rogers” phenomenon,^[6]^ because the numbers of metastatic lymph nodes could be influenced by those of lymph nodes harvested, for example, examination of a small number of lymph nodes might lead to underestimation of nodal status. Lee et al^[7]^ demonstrated that ∼46% of patients were misclassified by the 7th AJCC TNM staging system. Thus, the AJCC recommends the analysis of at least 16 lymph nodes for accurate evaluation of nodal stage in GC. Another option for nodal stage, also known as lymph node ratio (rN), which is the ratio of the number of positive lymph nodes to the total number of lymph nodes examined, has been proposed,^[8–11]^ but the result is identical to that of pN of the TNM system in patients classified as rN0. In other words, patients with pN0 cannot benefit from the rN staging system. In addition, Sun et al^[12]^ reported that patients with the same rN stage have different prognoses according to the total number of retrieved nodes. This opinion is based on the hypothesis that 3 positive lymph nodes of 3 retrieved nodes are not the same as 30 of 30. Moreover, the number of negative nodes is significantly related to the prognosis of patients with various types of malignancy.^[13–16]^ Deng et al^[15]^ demonstrated that the ratio of negative and positive lymph nodes is associated with prognosis prediction and concluded that this should be considered the optimal variable for evaluating the prognosis of GC in the clinic.

Recently, researchers proposed consideration of the log odds of positive lymph nodes (LODDS), which is defined as the log of the ratio of positive to negative nodes, as an alternative method for nodal staging and concluded that LODDS is superior to the pN or rN system in terms of predicting overall patient survival.^[12,17–21]^ They stated that LODDS is not influenced by the numbers of harvested lymph nodes and could predict survival even when fewer than 15 nodes are examined.

Despite these results, it is unclear whether LODDS is superior to the pN or rN system in terms of predicting the prognosis of GC patients who underwent radical gastrectomy with extended lymphadenectomy and had higher number of retrieved lymph nodes. Therefore, we investigated the validity of the LODDS system in Korean GC patients who underwent gastrectomy.

## 2 Materials and methods

### 2.1 Patients and data collection

The Institutional Review Board of Seoul St. Mary's Hospital approved the retrospective analysis of anonymous data involved in this study. The requirement for written informed consent was waived, but patient records were anonymized and deidentified prior to analysis. A total of 3929 patients who underwent gastrectomy for GC between January 1990 and December 2012 were enrolled. All patients underwent curative (R0) resection and extended lymphadenectomy. Patients who underwent palliative resection and those with known metastatic disease were excluded from the analysis.

Lymph node status was classified to the 7th AJCC N classification^[5]^ (pN0: no metastasis; pN1: 1 and 2 metastatic lymph nodes; pN2: 3–6 metastatic lymph nodes; pN3a: 7–14 metastatic lymph nodes; and pN3b: >15 metastatic lymph nodes) and the rN staging system (rN0: 0; 0 < rN1 ≤ 0.1; 0.1 < rN2 ≤ 0.2; 0.2 < rN3 ≤ 0.3; and rN4 > 0.3). The cutoff values of the rN system were determined by comparing 5-year survival rates according to rN with an initial interval of 0.1 and combining patients with similar prognoses (Table 1).

**Table 1:**
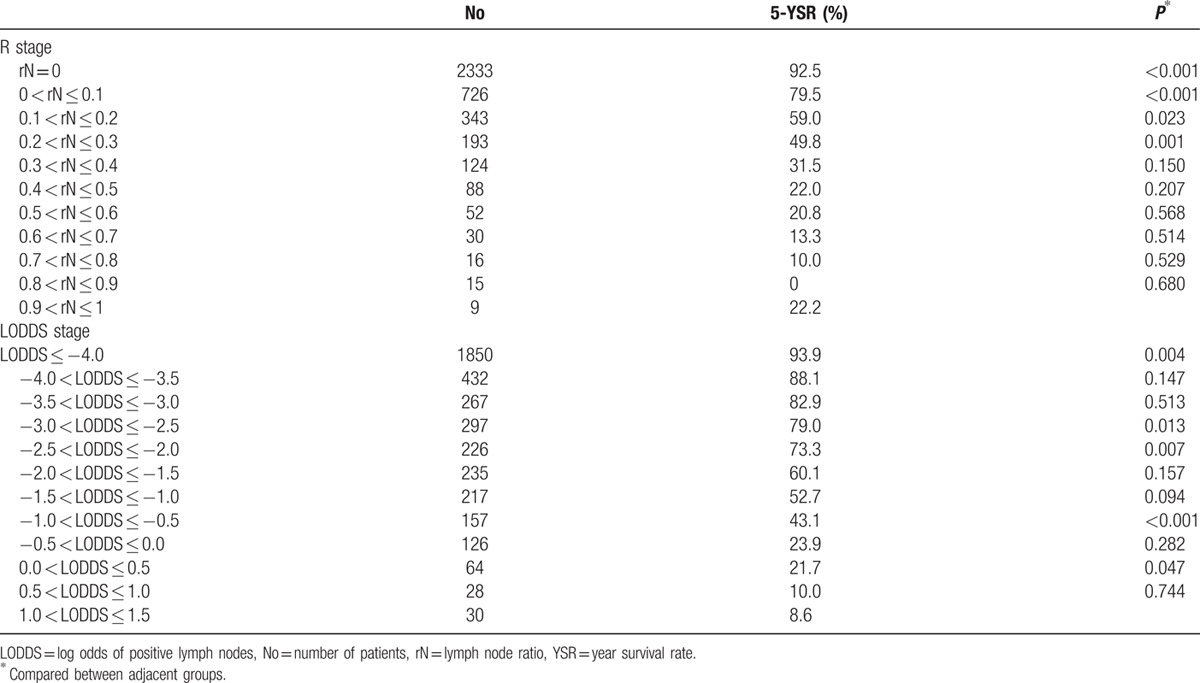
Overall survival rates according to the value of lymph node ratio and log odds of positive lymph nodes with the interval of 0.1 and 0.5.

LODDS was calculated by log (pnod + 0.5)/(tnod − pnod + 0.5) as in previous studies. We added 0.5 to avoid singularity. The cutoff values of the LODDS classification were determined by comparing 5-year survival rates (LODDS1 ≤ −4; −4 < LODDS2 ≤ −2.5; −2.5 < LODDS3 ≤ −2; −2 < LODDS4 ≤ −0.5; and LODDS5 > −0.5) (Table 1).

Overall survival rates were compared according to pN and rN classifications stratified by LODDS and according to LODDS stratified by pN and rN classifications.

Scatter plots of the association between LODDS and the absolute numbers of metastatic lymph nodes or rN were generated.

### 2.2 Definition of TRM and TLM stage

According to the 4 cutoff points defined in the methodology section, rN and LODDS stages were divided into 5 subgroups (rN0/LODDS1, rN1/ LODDS2, rN2/ LODDS3, rN3/ LODDS4, and rN4/LODDS5), corresponding to pN0, pN1, pN2, pN3a, and pN3b, respectively. The tumor-rN-metastasis (TRM) and tumor-LODDS-metastasis (TLM) systems were designed as combinations of the T stage, rN or LODDS stage, and M stage system including the 7th edition T stage and M stage of the TNM staging system. Then, we performed a Kaplan–Meier test of each staging system and a subgroup analysis was conducted only in patients of TNM stage IA according to the TLM staging system.

### 2.3 Statistical analysis

Continuous data were expressed as means ± standard deviation. Overall survival was estimated by the Kaplan–Meier method and differences in survival among the groups were investigated using the log-rank test. Survival duration was measured in months from the point of gastric resection. Any variables that were significant at a *P* value of less than 0.5 in Kaplan–Meier analysis were entered into a Cox proportional hazard model. Data analysis was performed using the SPSS software (version 12.0; SPSS, Chicago, IL). The critical *P* value for significance was set at 0.05.

## 3 Results

### 3.1 Demographic and clinicopathologic characteristics

The median follow-up time was 69.7 months. The baseline characteristics of enrolled patients are listed in Table 2. A total of 3929 patients with a median age of 59 years (range: 18–91) were identified. More than half of patients were male (66.7%, n = 2623). About half of the patients had tumors located in the lower 3rd of the stomach (52.4%, n = 2048).

**Table 2:**
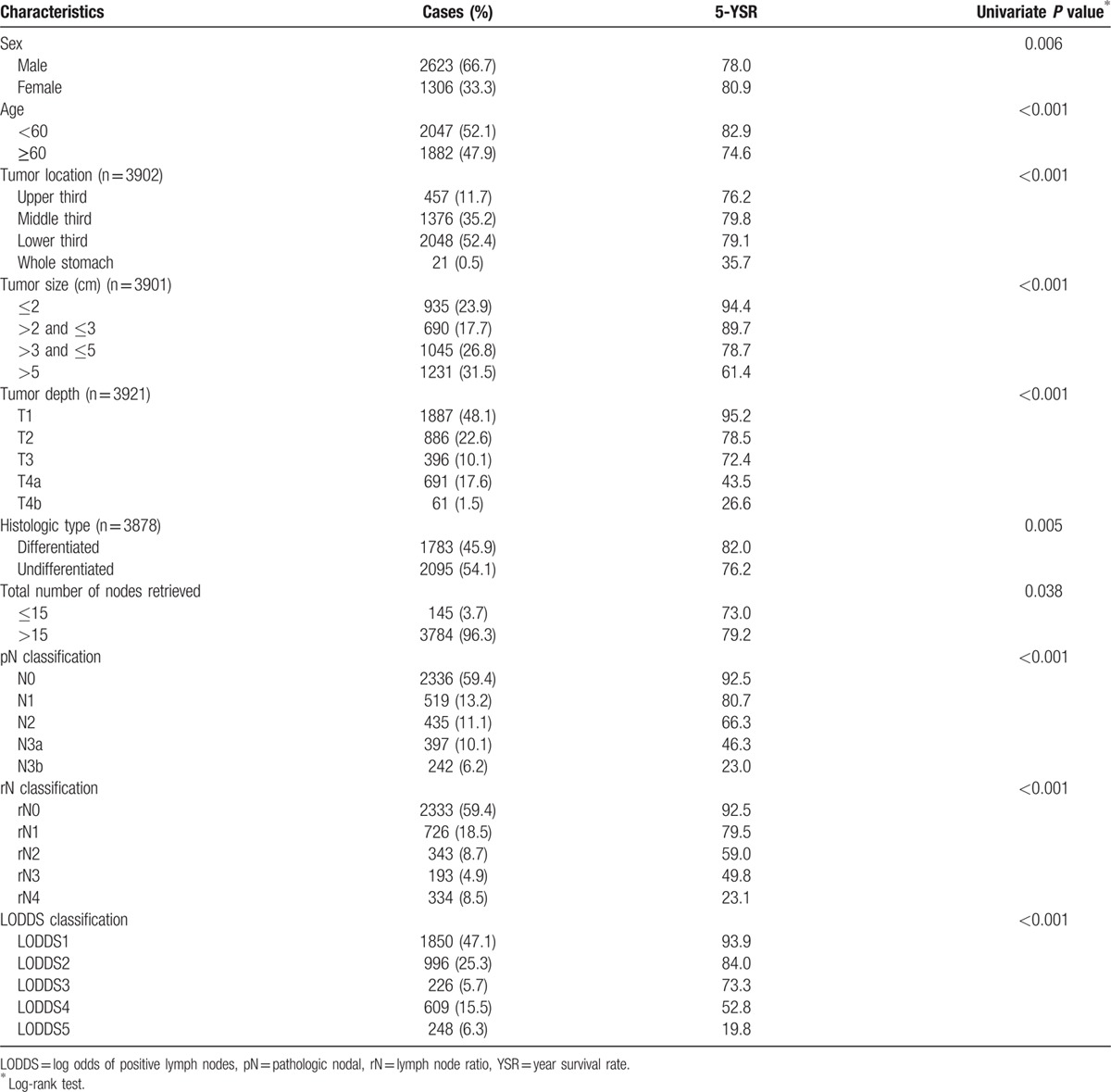
Demographics and survival analysis of 3929 patients.

On pathology, most patients were in stage pT1 (48.1%, n = 1887) and 22.6% of patients (n = 886) were stage pT2. Tumor size ranged from 0.1 to 34 cm, with a median of 3.7 cm and a mean of 4.4 ± 3.04 cm. Of 3878 patients, 1783 (45.9%) had differentiated-type histology. The mean numbers of retrieved lymph nodes and metastatic lymph nodes were 41.2 ± 16.5 and 3.22 ± 6.8, respectively. Based on the 7th edition of the AJCC staging system, patients with N0 were the most frequent (n = 2336, 59.4%).

Table 2 shows 5-year survival rates according to clinicopathologic characteristics and the 3 nodal staging systems. Male gender, age ≥60 years, tumor size, histologic type, tumor location, total number of nodes retrieved, tumor depth, and the aforementioned 3 nodal staging system were correlated significantly with 5-year survival. Multivariate Cox analysis adjusted for significant factors in the univariate analysis was used to assess the association of survival with pN stage (model 1), rN (model 2), and LODDS (model 3), separately. In models 1–3, all 3 staging systems were significantly correlated with survival (Table 3). Interestingly, the total number of lymph nodes retrieved was not predictive of survival in multivariate analysis, including the LODDS system (model 3).

**Table 3:**
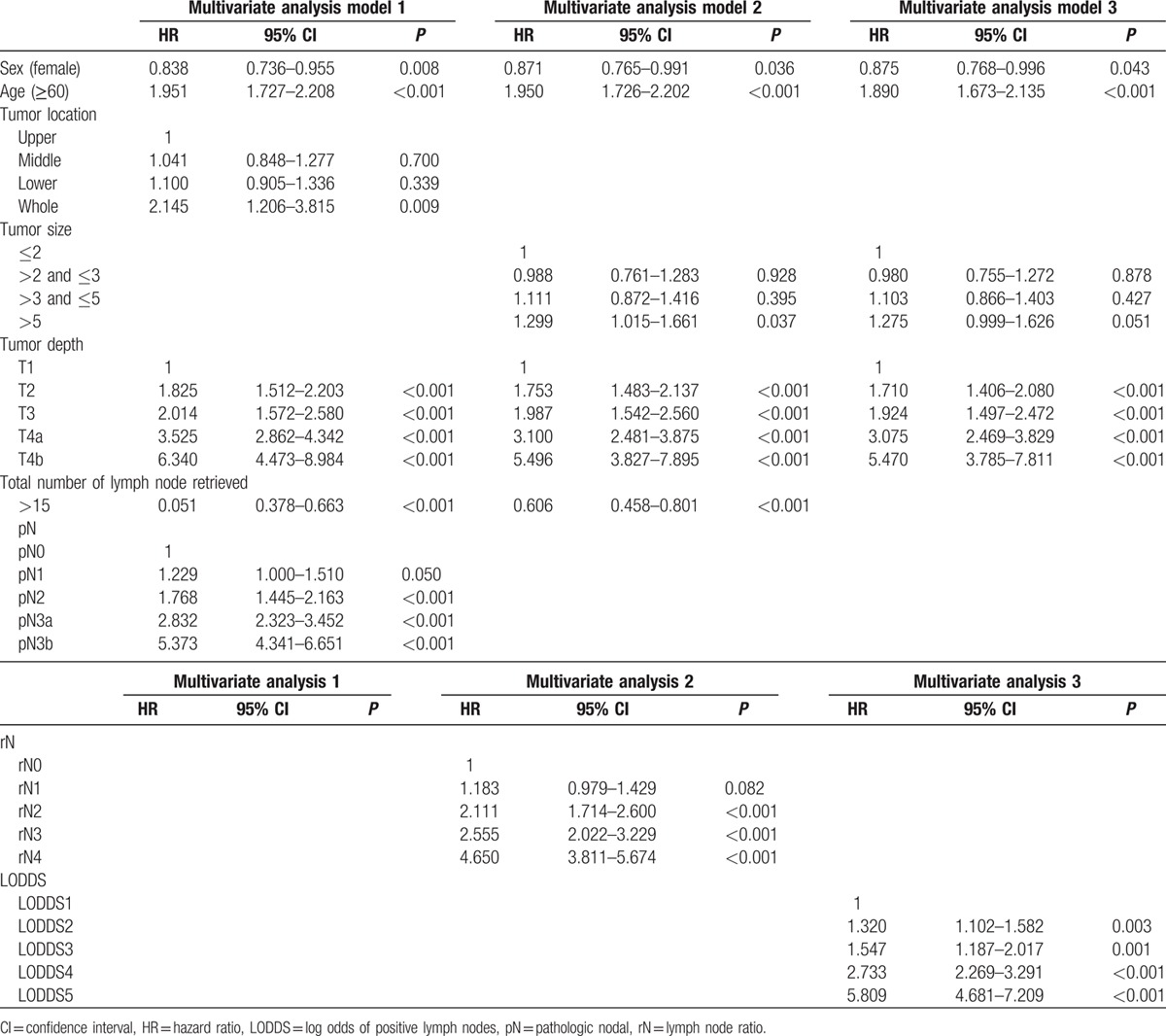
Three steps multivariate analysis of overall survival.

### 3.2 Distribution of LODDS according to metastatic lymph nodes and rN classifications

**Figure 1:**
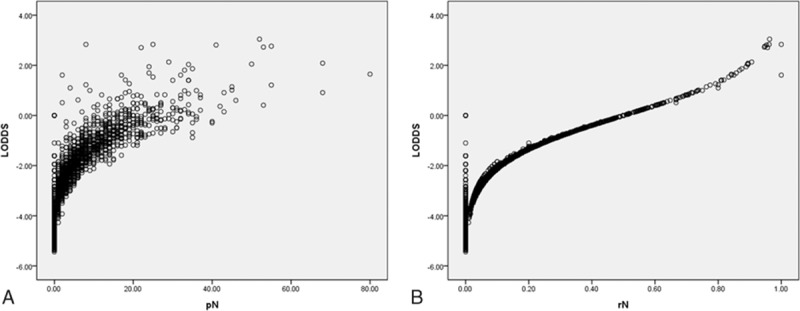
Scatter plots of the associations between LODDS and absolute numbers of metastatic lymph nodes (A) or rN (B). LODDS = log odds of positive lymph nodes, rN = lymph node ratio.

Figure 1 shows the relationship between LODDS and the other 2 systems. Spearman correlation test showed that LODDS correlated with pN and rN (data not shown), but the correlation was not linear. Particularly, patients with pN0 or rN1 were divided by LODDS classification, suggesting that the LODDS system could discriminate among patients with identical pN and rN classifications but with different prognoses. Previous studies demonstrated that these patients could benefit from the LODDS system because patients with identical pN0 or rN1 stages have different prognoses according to their total numbers of retrieved nodes.^[12,17–20]^

### 3.3 Correlation between total number of retrieved lymph node and the three staging systems

Spearman correlation test (Table 4) was used to evaluate the relations between the total numbers of retrieved lymph nodes and each staging system. Previous studies demonstrated that the number of harvested nodes influences the number of metastatic lymph nodes, but not LODDS. However, in the present study there were no significant correlations between each staging system and the number of retrieved lymph nodes (all coefficients │*r*│ < 0.3, *P* < 0.001).

**Table 4:**
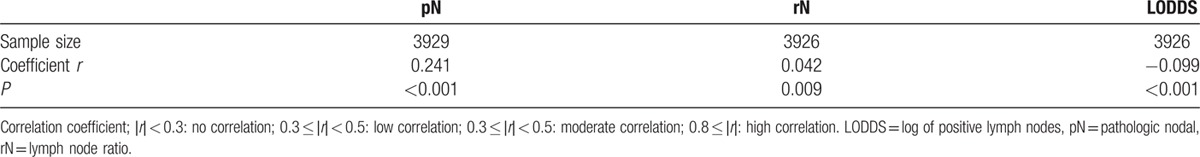
Correlation between of the number of lymph node retrieved and each staging system.

### 3.4 Comparison of overall survival using the 3 staging systems

**Figure 2:**
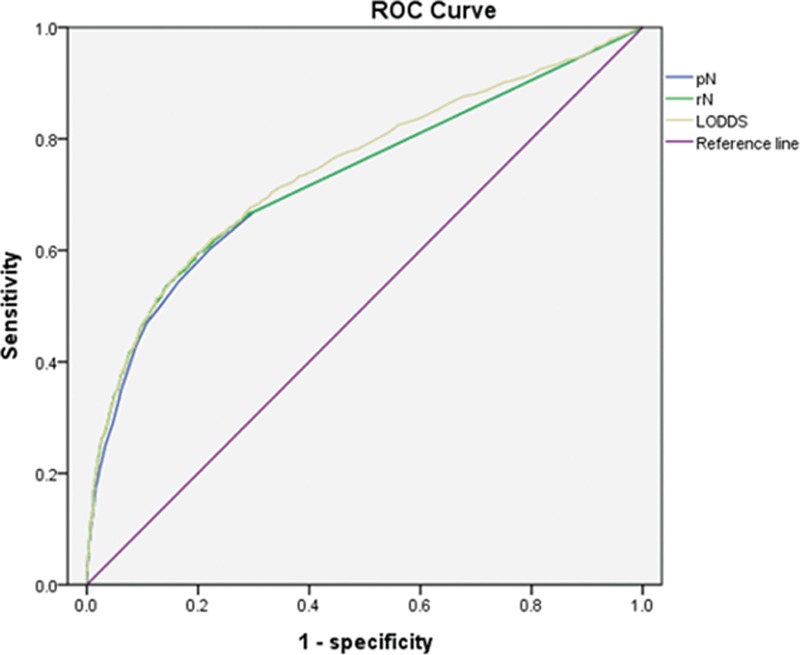
ROC curves of pN, rN, and LODDS for predicting survival. LODDS = log odds of positive lymph nodes, pN = pathologic nodal, rN = lymph node ratio, ROC = receiver operating characteristic.

Table 5 shows 5-year survival rates according to pN and rN classification stratified by LODDS. When stratified by the LODDS system, significant differences in survival were observed among patients in each pN and rN stage, with the exception of rN2. However, for patients with LODDS 4, survival rates were not homogenous when stratified by pN and rN classifications. The corresponding areas under the curve (AUC) for pN, rN, and LODDS were 0.728 (95% CI 0.709–0.747), 0.732 (95% CI 0.713–0.751), and 0.746 (95% CI 0.727–0.746), respectively, with no significant differences (*P *> 0.05) (Fig. 2).

**Table 5:**
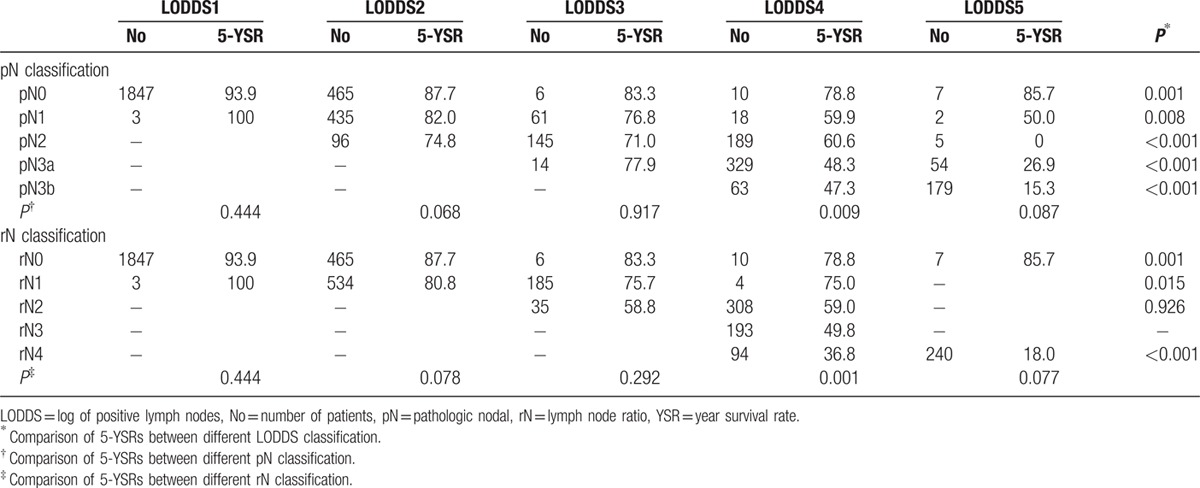
Overall survival rates with different pathologic nodal stage and lymph node ratio classifications stratified by the log odds of positive lymph nodes staging system.

### 3.5 TNM, TRM, and TLM

**Figure 3:**
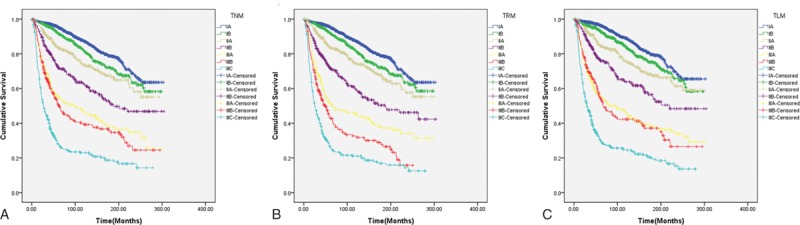
Efficacy of the TNM (A), TRM (B), and TLM (C) systems in terms of predicting survival of GC patients. GC = gastric cancer, LODDS = log odds of positive lymph nodes, rN = lymph node ratio, TLM = tumor LODDS metastasis, TNM = tumor node metastasis, TRM = tumor rN metastasis.

**Figure 4:**
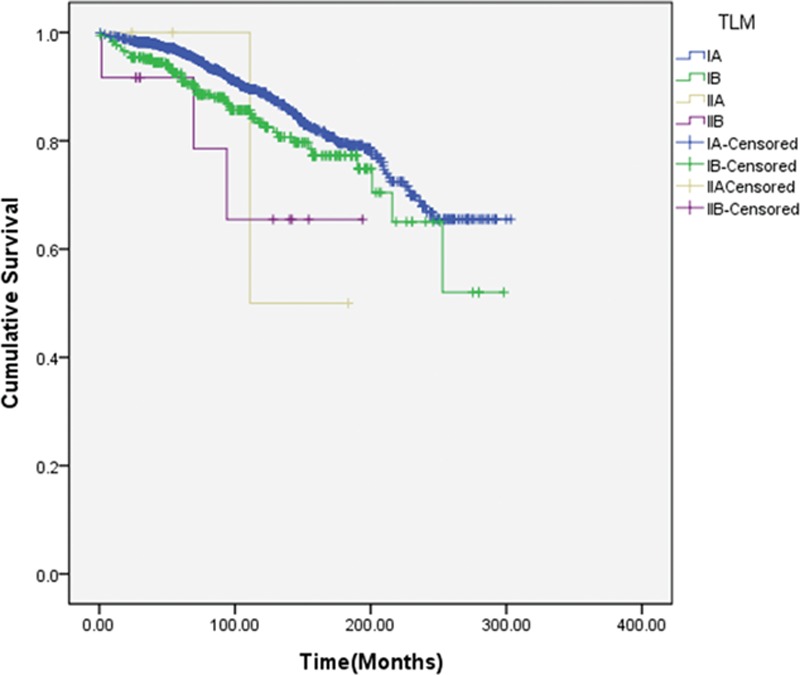
Efficacy of the TLM system in terms of predicting survival of TNM stage IA patients. LODDS = log odds of positive lymph nodes, TLM = tumor LODDS metastasis, TNM = tumor node metastasis.

We developed the TRM and TLM systems by replacing the pN stage with rN or LODDS. In these new systems, survival analysis using the Kaplan–Meier method showed that TRM or TLM was also effective in predicting the prognosis of patients with GC (Fig. 3). We examined the prognostic effect of the TLM system only in patients with TNM stage IA (Fig. 4). The TLM system enabled discrimination of prognosis among patients with TNM stage IA (*P* < 0.001). This subgroup included only 4 patients with TLM stage IIA; 3 of these 4 patients were censored during the study period.

## 4 Discussion

Recently, many studies have attempted to develop novel staging systems, such as the ratio-based (rN)^[8–11]^ and LODDS^[12,17–21]^ classification systems, the latter of which was employed in a study of breast, colorectal, and GC.^[22–25]^ However, controversy regarding whether these new classification systems are superior to the number-based classification system remains.

Odds are a numerical expression that predicts probability and are usually used in gambling and statistics. The hypothesis that LODDS is superior to rN systems is based on its ability to discriminate survival among patients with the same rN. Theoretically, if the rNs of 2 patients with different numbers of harvested nodes are identical, the odds would also be identical, for example, if the rN is 0.5, odds would be 1 regardless of the number of lymph nodes retrieved. The reason that LODDS could discriminate survival among patients with same the rN is that 0.5 was added to both the denominator and numerator to avoid singularity. In this manner, negative lymph nodes are included in assessment of prognosis, even in patients with rN0.

The most important reason for developing another lymph node staging system beyond the absolute number of metastatic nodes (pN) is that stage migration usually occurs due to the small number of lymph nodes retrieved. In a recent study with 2935 Korean patients, the mean number of examined lymph nodes was 42.^[26]^

A comparison study showed that only 3% of Korean patients had fewer than 15 retrieved lymph nodes, compared to 22% of US patients.^[27]^ In the current study, we evaluated whether the rN or LODDS system would be superior to the conventional pN system in Korean patients who underwent extensive lymph node dissection.

Univariate and multivariate analysis using the Kaplan–Meier method showed that the pN, rN, and LODDS systems were significant prognostic factors in terms of survival. To determine whether the LODDS classification system is superior to the other systems, we analyzed survival of patients with each pN and rN classification stratified by LODDS. When stratified by LODDS, significant differences were observed for patients with each pN and rN, with the exception of rN2; however, survival in patients with LODDS 4 was heterogeneous when stratified by the pN and rN classifications. These results suggested that the use of LODDS may facilitate more accurate staging for prognosis and that it is superior to other systems, although the receiver operating characteristic (ROC)-AUC curve showed that the 3 staging systems did not differ significantly in terms of predicting survival. The results of the present study are consistent with those of previous studies that focused on patients with an inadequate number of lymph nodes harvested.

This study has several limitations that should be considered. On the one hand, it was a nonrandomized, retrospective, single-center study. On the other hand, the optimal cutoff points could only predict prognosis. Due to the lack of a separate validation set, the predictive power of the LODDS system could not be evaluated. Therefore, large-scale and prospective multicenter studies are needed. However, the mean number of retrieved lymph nodes in our study was greater than those used in previous studies, and the proportion of patients with <16 harvested lymph nodes was 3.7%, which was lower than in previous studies (Table 6).

**Table 6:**
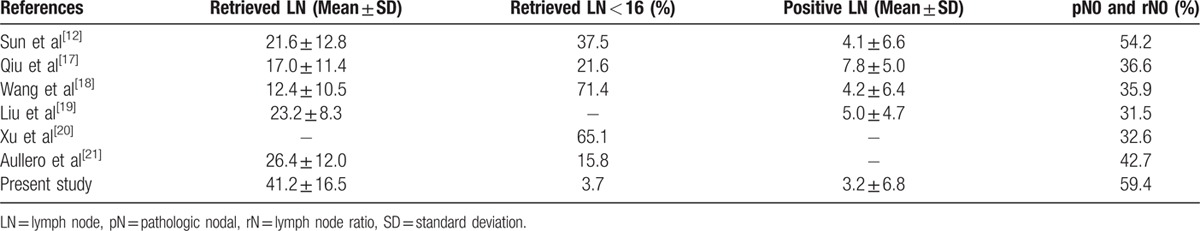
Comparison of lymph node status between present and previous study.

In conclusion, LODDS is independently and significantly associated with overall survival in patients with GC, and its prognostic value is superior to those of two other lymph node staging systems in Korean patients. LODDS may be incorporated into the GC staging system if these results are confirmed by other studies.
